# Can treatment with Cocculine improve the control of chemotherapy-induced emesis in early breast cancer patients? A randomized, multi-centered, double-blind, placebo-controlled Phase III trial

**DOI:** 10.1186/1471-2407-12-603

**Published:** 2012-12-17

**Authors:** David Pérol, Jocelyne Provençal, Anne-claire Hardy-Bessard, David Coeffic, Jean-Phillipe Jacquin, Cécile Agostini, Thomas Bachelot, Jean-Paul Guastalla, Xavier Pivot, Jean-Pierre Martin, Agathe Bajard, Isabelle Ray-Coquard

**Affiliations:** 1 Centre Léon Bérard, 28 rue Laennec, Lyon Cedex 08, 69373, France; 2 Centre hospitalier de la région d’Annecy, 1 avenue de l’hôpital, Annecy, BP90074, 74374, France; 3 Clinique armoricaine de Radiologie, 21 rue du Vieux Séminaire, Saint Brieuc, 22 000, France; 4 UMGEC, Service Institut Daniel Hollard, 12 Rue Docteur Calmette, Grenoble, 38028, France; 5 Institut de cancérologie de la Loire, 108, avenue Albert-Raimond, Saint-Priest-en-Jarez, 42270, France; 6 Centre hospitalier Général, Chambéry, BP1125, 73011, France; 7 Centre Hospitalier Universitaire, Boulevard Fleming, Besançon, 25030, France; 8 Hôpital Jean Mermoz, 55 avenue Jean Mermoz, Lyon, 69008, France; 9 Centre Léon Bérard, 28 rue Laennec, Lyon, 69008, France

**Keywords:** Early breast cancer, Adjuvant chemotherapy, Homeopathy, Nausea and vomiting, Quality of life

## Abstract

**Background:**

Chemotherapy induced nausea and vomiting (CINV) remains a major problem that seriously impairs the quality of life (QoL) in cancer patients receiving chemotherapy regimens. Complementary medicines, including homeopathy, are used by many patients with cancer, usually alongside with conventional treatment. A randomized, placebo-controlled Phase III study was conducted to evaluate the efficacy of a complex homeopathic medicine, Cocculine, in the control of CINV in non-metastatic breast cancer patients treated by standard chemotherapy regimens.

**Methods:**

Chemotherapy-naïve patients with non-metastatic breast cancer scheduled to receive 6 cycles of chemotherapy including at least three initial cycles of FAC 50, FEC 100 or TAC were randomized to receive standard anti-emetic treatment plus either a complex homeopathic remedy (*Cocculine*, registered in France for treatment of nausea and travel sickness) or the matching placebo (NCT00409071 clinicaltrials.gov). The primary endpoint was nausea score measured after the 1^st^ chemotherapy course using the FLIE questionnaire (Functional Living Index for Emesis) with 5-day recall. Secondary endpoints were: vomiting measured by the FLIE score, nausea and vomiting measured by patient self-evaluation (EVA) and investigator recording (NCI-CTC AE V3.0) and treatment compliance.

**Results:**

From September 2005 to January 2008, 431 patients were randomized: 214 to *Cocculine* (C) and 217 to placebo (P). Patient characteristics were well-balanced between the 2 arms. Overall, compliance to study treatments was excellent and similar between the 2 arms. A total of 205 patients (50.9%; 103 patients in the placebo and 102 in the homeopathy arms) had nausea FLIE scores > 6 indicative of no impact of nausea on quality of life during the 1^st^ chemotherapy course. There was no difference between the 2 arms when primary endpoint analysis was performed by chemotherapy stratum; or in the subgroup of patients with susceptibility to nausea and vomiting before inclusion. In addition, nausea, vomiting and global emesis FLIE scores were not statistically different at any time between the two study arms. The frequencies of severe (Grade ≥ 2) nausea and vomiting were low in our study (nausea: P: 17.6% vs C: 15.7%, p=0.62; vomiting: P: 10.8% vs C: 12.0%, p=0.72 during the first course).

**Conclusion:**

This double-blinded, placebo-controlled, randomised Phase III study showed that adding a complex homeopathic medicine (Cocculine) to standard anti-emetic prophylaxis does not improve the control of CINV in early breast cancer patients.

## Background

Chemotherapy induced nausea and vomiting (CINV) are among the most severe and feared collateral effects of chemotherapy [1-3] associated with a significant deterioration in quality of life (QoL) and patients’ ability to carry out daily activities [4,5]. Poor QoL can influence the patient’s willingness to continue with and successfully complete cancer treatment. Therefore, it is essential to prevent and treat these side effects optimally to maximize QoL and to encourage patient compliance [5,6].

Over the past few years, selective serotonin type 3 receptor (5-HT3) antagonists and the neurokinin-1 (NK-1) antagonist aprepitant have substantially improved the management of acute CINV (occurring within 24 hours of chemotherapy) and to a lesser extent delayed CINV (occurring more than 24 hours post-chemotherapy) [7-12]. Nevertheless, 75% percent of patients with nausea and 50% of those with vomiting reported a negative impact on the performance of daily living activities when queried with the Functional Living Index–Emesis (FLIE) questionnaire despite modern prophylactic anti-emetic treatment [13]. To date, CINV remains a significant problem contributing to patient withdrawal from potentially curative chemotherapy [5,8-10].

In an attempt to alleviate collateral effects of cancer therapies, complementary and alternative medicines are increasingly used by cancer patients [14,15]. In an European survey, 35.9% of cancer patients have reported using some form of complementary or alternative medicines (CAM) to reduce cancer treatment-related adverse events (AEs) [15]. Homeopathy was in the top five of the most commonly used CAM in 7 out of 14 European countries [15]. Overall, homeopathic approaches are used by cancer patients to alleviate their pain resulting from the disease itself or from conventional anti-cancer treatment [15]. Homeopathic medicines efficacy have been studied in the treatment of adverse effects of radiotherapy and chemotherapy in breast cancer patients. However, large, randomized, placebo-controlled trials with powered statistical analysis are needed to generate evidence-based data on the value of complementary medicine.

Different homeopathic practices coexist: the “classical” or “individualised” homeopathy using single homeopathic medicine that is prescribed according to the individual’s condition and history, (ii) the “clinical” homeopathy that uses the same homeopathic medicine for a group of patients with the same disease and (iii) the “complex” homeopathy that uses more than one homeopathic medicine, in a fixed combination or concurrently, for a particular condition. Cocculine is a homeopathic medicinal product registered in France for treatment of nausea and travel sickness composed of 4 homeopathic components (Cocculus indicus 4 CH, Tabacum 4 CH, Nux vomica 4 CH, Petroleum 4 CH produced by Boiron, France, according to European Pharmacopoeia). A recent study has demonstrated that *Cocculine* has a potential interest for the management of CINV with a 30% reduction of nausea under *Cocculine* treatment compared to placebo in breast cancer patients treated by chemotherapy (overall n=80: incidence of nausea 61.5% in *Cocculine* arm versus 87.5% with placebo [16]). The main objective of the present study was to evaluate if this complex homoepathic medicine can improve the control of CINV in non-metastatic breast cancer patients in a large, randomized, multicenter Phase III trial.

## Methods

### Patients

Eligible women patients were chemotherapy-naive adults with non metastatic, histologically proven breast cancer and an Eastern Cooperative Oncology Group performance status (ECOG PS) of ≤ 2, scheduled to receive 6 cycles of standard adjuvant chemotherapy. The first 3 cycles were required to be FAC50 (5-Fluoruracil 500 mg/m [2] + adriamycin [doxorubicin] 50 mg/m [2] + cyclophosphamide 500 mg/m [2]), FEC100 (5-Fluoruracil 500 mg/m [2] + epirubicin 100 mg/m [2] + cyclophosphamide 500 mg/m [2]) or TAC (Taxotere [Docetaxel] 75 mg/m [2] + adriamycine 50 mg/m [2] + cyclophosphamide 500 mg/m [2]). Patients with previous malignancies (except those in complete remission for more than 5 years), contraindications to corticoids or 5-HT3 receptor antagonists, or prior treatment with Cocculine or other anti-emetics within the previous 15 days were ineligible. Pregnant or lactating patients, those who could not be followed up for social, geographical, familial or psychological reasons or unavailability by phone were also excluded. The protocol was approved by a French ethical committee (CPP Sud Est IV) and registered with clinicaltrials.gov as NCT00409071. All included patients have signed an inform consent form before study entry.

### Randomisation and blinding

Randomisation was conducted centrally via a computer-generated system, using permuted blocks of four patients (the Jadad/Oxford score was ≥ 3/5). Patients were stratified by participating centre and type of chemotherapy regimen (FAC50 or FEC100 versus TAC). Allocation list was generated by the study statistician before the beginning of the study. Investigators asked the coordinating center by fax for a treatment allocation number. Both investigator and patient remained blind to the assignment of individuals to either active treatment or placebo throughout the study.

### Study treatments

The trial used a randomized, multicenter, double blind phase III design in which patients were randomly assigned to receive placebo or Cocculine (Cocculine^®^, Boiron- France) plus standard anti emetic treatment. Cocculine is a complex of four active elements incorporated in the same tablets. The placebo tablets were identical seemingly in the active tablets (packaging, colour, shape…). The placebo tablets were inert and contained only (Saccharose (75%), lactose (24%), and Magnesium stearate (1%)) without any homeopathic components. All patients received a box containing six * two blister packs of ten tablets corresponding to the treatment number allocated at randomisation (Cocculine or matching placebo). Patients had to return boxes and tablet blisters to the investigator at the end of treatment. Two tablets were to be taken in the evening before chemotherapy, 6 on the day of chemotherapy and 4 tablets the next day (see Table 1). A standard anti-emetic treatment was given to patient: ondansetron 8 mg (or granisetron 3 mg in case of intolerance to ondansetron) and methylpredinosolone 80 mg. While this trial was being conducted, consensus guidelines were updated to recommend the use of a three-drug combination of steroids plus 5-HT3 receptor antagonist plus the NK-1 receptor antagonist aprepitant [7,17]. At the time of patient enrolment, aprepitant was not recommended and so was not used during the study. The study flow chart is presented in Table 1.

**Table 1:** Study flow-chart (per chemotherapy cycle)

	**Day-1**	**Day 1**			**Day 2**			**Day3**	**Day4**	**Day 5**	**Day 6**
Tablets of Cocculine or placebo	Evening	Morning	Noon	Evening	Morning	Noon	Evening	Morning			
Antiemetic treatment	2	2	2	2	2	2					
Chemotherapy	P^1^ (TAC)	S^2^ + M^3^		S^4^ + P^1^	S^4^ + P^1^		S^4^ + P^1^	S^4^ + P^1^			
Phone call	X*		X							X**	
FLIE questionnaire											
Patients diary											X
				X			X	X	X	X	

Po: per os; IV: intraverious; P: Prednisolone; M: Methyprednisolone; S: Ondansetron.

^1^: 60 mg po (TAC only); ^2^: 8 mg IV; ^3^: 60mg IV; ^4^: 8 mg po; *: all patients; **: on patients’ request.

### Assessment

The primary objective was to evaluate the efficacy of Cocculine versus Placebo when added to conventional corticoid plus setron prophylaxis in the control of chemotherapy-induced nausea during the 1^st^ cycle of chemotherapy. The secondary objectives were to evaluate the efficacy of Cocculine during the 2^nd^ and 3^rd^ cycles, the contribution of Cocculine in the treatment of nausea and vomiting, and compliance to Cocculine treatment. To evaluate the impact of homeopathy remedy in the control of CINV based on patients’ assessment: a self-assessment booklet composed of the FLIE questionnaire [18,19] and a specific diary were given to patients. In addition, CINV were also evaluated based on investigators’ assessment using the CTCAE V3.0 grading scale.

The self–administered FLIE questionnaire is composed of two dimensions (nausea and vomiting) each with 9 items. Each item consists of a horizontal Visual Analogical Scale (VAS) of 100 mm graduated from 1 (a lot) to 7 (not at all). The first question in each domain asks the patient to rate how much nausea or vomiting she has experienced over the past 5 days. The remaining eight questions specifically address the impact of CINV on daily activities (i.e. physical abilities, social and emotional function) [18,19]. No or minimal impact on daily life was defined as an average FLIE item score > 6. Patients completed the FLIE questionnaire on Day 6 of the first 3 chemotherapy cycles.

Patients were also provided with a daily diary to record i) intake of study drug ii) the occurrence and intensity of nausea during the first 24 hours and over days 2 to 5 following chemotherapy and the number of vomiting episodes, and iii) use of any rescue antiemetic medications. All patients were contacted by telephone on the day before the start of chemotherapy and (if they requested this) on day 5 of the first cycle to ensure that the diary and FLIE questionnaire had been completed accurately. In addition, the incidence and severity of AEs (NCI-CTCAE v3.0, ctep.cancer.gov/protocolDevelopment/electronic…/docs/ctcaev3.pdf) including nausea and vomiting were recorded by the investigator at the end of each chemotherapy cycle. Data were collected until either the cessation of chemotherapy or the administration of a maximum of 6 cycles of treatment.

### Statistical analysis

The study was powered to detect a 0.5 point difference between treatment arms in the FLIE nausea score after the 1^st^ CT cycle. Accepting a two-sided type I error of 5% and a type II error of 15%, 198 patients were required per group. The statistical analysis was performed by intent-to-treat.

The primary endpoint was the mean of 9 first FLIE items (at least 5 out of 9 items had to be completed). Scores were compared between the two arms using the non parametric Mann–Whitney U test. The number of patients with a mean score > 6 versus the number of patients with a mean score ≤ 6 were compared using Fisher’s exact test.

The emesis score after the 1^st^, 2^nd^ and 3^rd^ chemotherapy cycles and the nausea score after the 2^nd^ and 3^rd^ chemotherapy cycles were calculated in order to evaluate the efficacy of Cocculine over the first 3 CT cycles.

Vomiting frequency reported on the VAS during the 1^st^, 2^nd^ and 3^rd^ CT courses was compared between the two arms by a Pearson’s chi-square test (or a Fisher’s exact test, if appropriate).

Compliance was compared between the 2 arms using the diary and by counting the amount of drug that remained in its packaging. AEs were compared between the two arms over the 6-cycle period with particular attention to nausea or vomiting.

All analyses were performed using the SAS software, version 9.1 (SAS Institute, Cary, NC).

## Results

### Assignment of patients and treatment compliance

**Figure 1:**
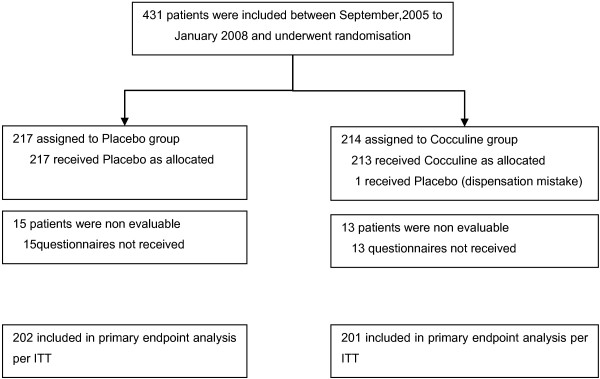
CONSORT diagram.

From September 2005 to January 2008, 431 non metastatic breast cancer patients were enrolled in 8 centres; 214 patients were randomly assigned to Cocculine (C) and 217 to placebo (Figure 1). A total of 266 patients (61.7%) were in the FAC/FEC stratum and 165 (38.3%) in the TAC stratum. Major patient characteristics at baseline are detailed in Table 2. The two arms were well-balanced, with no statistically significant differences between them. Median age was 52.8 years (range 20–74); 237 patients (55.6%) were T1 a-b-c and 258 patients (60.6%) were pN+; and 35.3% of the patients had susceptibility to nausea or vomiting. Treatment compliance as estimated using patient diary and tablet count was largely acceptable: according to the diary, 71% of patients had taken the study drug per protocol, and this figure was 81% according to the count of remaining tablets (data not shown). Compliance was similar between arms. A total of 263 patients (63.2%) had taken standard anti-emetic treatment per protocol during cycle 1. A total of 384 patients (90.8%) had received chemotherapy for the entire study period but 146 patients (34.5%) had to delay CT (mainly due to haematological toxicity) and 34 patients (8.0%) had at least one dose reduction over the 6-cycle period. The number of delays and/or dose reductions was similar between the two arms (data not shown). There was no impact of Cocculine treatment on compliance to chemotherapy regimen. Of note, there was no statistical difference in the use of concomitant anti-emetic rescue medication between the 2 study arms at any time during the study period (data not shown).

**Table 2:** Patient characteristic

**Patient characteristics**	**Placebo**	**Cocculine**	**All patients**	**p**
Total	217	214	431	
**Age,** years				0.56*
Median (min-max)	52.8 (20–74)	53.3 (30–74)	52.8 (20–74)	
**Delay from surgery (days)**				
Median (min-max)	27.50 (4.00–70.0)	27.0 (1.0–72.0)	27.0 (1.0–72.0)	0.90*
**ECOG Performance Status**	n = 197	n = 198	n = 395	0.64†
0, *n (%)*	177 (89.8)	175 (88.4)	352 (89.1)	
1, *n (%)*	20 (10.2)	23 (11.6)	43 (10.9)	
**Nausea and vomiting susceptibility**	n = 188	n = 189	n = 377	0.47†
Yes *n (%)*	63 (33.5)	70 (37.0)	133 (35.3)	
**pT grade**	n = 215	n = 211	n = 426	1.00††
T1 a-b-c, *n(%)*	119 (55.3)	118 (55.9)	237 (55.6)	
T2, *n(%)*	87 (40.5)	85 (40.3)	172 (40.4)	
T3, *n(%)*	7 (3.3)	7 (3.3)	14 (3.3)	
T4, *n(%)*	2 (0.9)	1 (0.5)	3 (0.7)	
**pN grade**	n = 213	n = 213	n = 426	0.84†
N-, *n(%)*	85 (39.9)	83 (39.0)	168 (39.4)	
N+, *n(%)*	128 (60.1)	130 (61.0)	258 (60.6)	

*: Mann–Whitney U test; † : Person’s chi-square test; †† : Fischer test.

### CINV assessed by patients using FLIE scores [Table 3] or daily diaries [Table 4]

**Table 3:** Impact of nausea on quality of life during cycle 1 of chemotherapy: nausea dimension of the FLIE score in ITT population

	**Placebo**	**Cocculine**	**All patients**	**p**
**All patients**	n=217	n=214	n=431	
missing data**	*15*	*13*	*28*	
*number of patients evaluable*	*202*	*201*	*403*	
**Quantitative**				0.84*
Median nausea Score (Min-Max)	6.02 (1.11 – 7)	6.07 (1.22 – 7)	6.02 (1.11 – 7)	
Mean ± sd	5.43 ± 1.57	5.45 ± 1.57	5.44 ± 1.57	
**Qualitative**				
Patients with no impact of nausea on daily life (i.e. a median score > 6), n (%)	103 (51.0)	102 (50.7)	205 (50.9)	0.96†
**Stratum FAC-FEC**	n=135	n=131	n=266	
missing data **	*9*	*7*	*16*	
*number of patients evaluable*	*126*	*124*	*250*	
***Quantitative***				
Median nausea score (min-max)	5.93 (1.11–7)	6.08 (1.65–7)	6.03 (1.11–7)	0.69*
Mean ± sd	5.37 (1.66)	5.48 (1.52)	5.42 (1.49)	
***Qualitative***				
Patients with no impact of nausea on daily life (i.e. mean score > 6), n (%)	63 (50.0)	63 (50.8)	126 (50.4)	0.90†
**Stratum TAC**	n=82	n=83	n=165	
missing data**	*6*	*6*	*12*	
*number of patients evaluable*	76	77	153	
***Quantitative***				
Median nausea score (min-max)		6.07 (1.90-7)		6.02 (1.22-7)
Mean ± sd	5.54 (1.43)	5.39 (1.66)	5.47 (1.55)	
***Qualitative***				
Patients with no impact of nausea on daily life (i.e. mean score > 6), n (%)	40 (52.6)	39 (50.6)	79 (51.6)	0.81†
**Patients with susceptibility to NV (Yes)**	n=61	n=66	n=127	
missing data**	*2*	*4*	*6*	
*number of patients evaluable*	*59*	*62*	*121*	
***Quantitative***				
Median nausea score (min-max)	5.85 (1.11–7)	5.81 (1.22–7)	5.83 (1.11–7)	0.36*
Mean ± sd	5.29 (1.66)	5.04 (1.71)	5.16 (1.69)	
***Qualitative***				
Patients with no impact of nausea on daily life (i.e. mean score > 6), n (%)	30 (49.2)	28 (42.4)	58 (45.7)	0.45†

*: Mann–Whitney U test †: Person’s chi-square test.

**: missing data i.e. number of patients with more than 5 missing items in the FLIE questionnaire.

n: number of patients.

**Table 4:** **Patient self -evaluation of nausea and vomiting during the 1**^**st**^**CT cycle (ITT population)**

	**Placebo n = 217**	**Cocculine n = 214**	**All patients n = 431**	**p**
**Nausea frequency**				0.59†
*missing data*	*16*	*19*	*35*	
*number of patients evaluable*	*201*	*195*	*396*	
Number of patients with at least 1 episode of nausea, n (%)	164 (81.6)	155 (79.5)	319 (80.6)	
**Nausea severity**				0.61*
*missing data*	*16*	*19*	*35*	
*number of patients evalubale*	*201*	*195*	*396*	
Median (min-max)	0.56 (0–8.5)	0.58 (0–8.3)	0.56 (0–8.5)	
**Vomiting frequency**				0.56†
*missing data*	*31*	*19*	*50*	
*number of patients evaluable*	*186*	*170*	*356*	
Number of patients with at least 1 episode of vomiting, n (%)	51 (27.4)	42 (24.7)	93 (26.1)	
**Vomiting severity**				
*missing data*	*22*	*25*	*47*	0.44*
*number of patients evaluable*	*195*	*189*	*384*	
Median (min-max)	0.00 (0–7.60)	0.00 (0–5.0)	0.00 (0–7.60)	

*: Mann–Whitney U test †: Person’s chi-square test.

In total, 403 of 431 patients (93.5%) were assessable for the primary endpoint (FLIE nausea score during 1^st^ CT course): 28 patients were non evaluable, 15 in the placebo and 13 in the Cocculine arms (Table 3). Non-assessable patients were accounted for 7 who withdrew consent, 17 from whom questionnaires were not received, and 4 who returned questionnaires that were not evaluable.

Using the FLIE questionnaire, nausea scores after the 1^st^ chemotherapy cycle were 6.02 and 6.07 for placebo and Cocculine arms, respectively (p = 0.84). A total of 205 patients (50.9%; 103 patients in the placebo and 102 in the Cocculine arms) had scores > 6 indicative of no impact of nausea on quality of life. FLIE analysis results are reported in Table 3. There was no difference between the 2 arms when analysis was performed by chemotherapy stratum; and there was no difference in the subgroup of patients with known susceptibility to nausea and vomiting (Table 3).

Nausea, vomiting and global emesis FLIE scores were not statistically different at any time between the two arms (data not shown).

Based on daily diaries, the intensity of nausea reported by patients over the first 3 cycles of chemotherapy was very low (median nausea severity: 1^st^ cycle: 0.56 [P] vs. 0.58 [C], p = 0.61 [Table 4]; 2^nd^ cycle: 0.52 [P] vs. 0.60[C], p=0.36; and 3^rd^ cycle: 0.93 [P] vs. 0.40 [C]; p=0.45, data not shown) with no significant difference between the two arms. Patient had few vomiting episodes of low intensity (medians were 0 for the 3 cycles). More patients reported vomiting episodes during the 3^rd^ CT course in the placebo (34.2%) than in the Cocculine arms (23.4%), p = 0.03 (data not shown). However, these observations were not maintained over the 4, 5 and 6^th^ cycles of CT and had no impact on FLIE vomiting score.

### CINV assessed by investigators (NCI –CTCAE V3.0 grading)

Based on investigators assessments using CTCAE V3.0, nausea occurred in 51.1% versus 47.4% of the patients treated with placebo and Cocculine respectively (p = 0.48) during the 1^st^ CT cycle (Table 5), in 42.3% (P) versus 48.9% (C) (p = 0.21) during the 2^nd^ CT cycle; and in 43.2% (P) versus 45.8% (C) (p = 0.63) during the 3^rd^ CT cycle (data not shown). No significant differences were noted during cycles 4 to 6.

**Table 5:** **Frequency of nausea and vomiting AEs during 1**^**st**^**cycle of chemotherapy**

**Frequency of nausea and vomiting AE (all grade) during 1**^**st**^**cycle of chemotherapy**				
	**Placebo n = 217**	**Cocculine**^®^**n = 214**	**All n = 431**	**p** †
*Missing data*	*39*	*20*	*59*	
*Number of patients evaluable*	*178*	*194*	*372*	
Number of patient with at least 1 AE nausea (%)	91 (51.1)	92 (47.4)	83 (49.2)	0.48
*Missing data*	*39*	*20*	*59*	
*Number of patients evaluable*	*178*	*194*	*372*	
Number of patient with at least 1 AE vomiting, n (%)	35 (19.7)	41 (21.1)	76 (20.4)	0.72
**Frequency of severe nausea and vomiting AE (Grade ≥ 2) during 1**^**st**^**cycle of chemotherapy**				
	**Placebo n = 217**	**Cocculine n = 214**	**All n = 431**	**p** †
*Missing data*	*47*	*29*	*76*	
*Number of patients evaluable, n*	*170*	*185*	*355*	
Number of patient with at least 1 severe nausea AE (i.e. Grade ≥2), n (%)	30 (17.6)	29 (15.7)	59 (16.6)	0.62
*Missing data*	*41*	*22*	*63*	
*Number of patients evaluable*	*176*	*192*	*368*	
Number of patient with at least 1 severe vomiting AE (i.e. Grade ≥ 2), n (%)	19 (10.8)	23 (12.0)	42 (11.4)	0.72

†Person’s chi-square test.

Frequency of vomiting was also similar between the 2 arms: 19.7% versus 21.1% (p = 0.72) for Placebo and Cocculine respectively during the 1^st^ CT cycle (Table 5); 13.7% (P) versus 15.2% (C) (p = 0.7) during the 2^nd^ CT cycle; and 22.2 (P) versus 16.9% (C) (p = 0.22) during the 3^rd^ CT cycle (data not shown). The incidence of severe nausea and vomiting (i.e. Grade ≥ 2) reported by the investigators during the 1^st^ CT cycle was also similar between the 2 treatment groups (Table 5): severe nausea occurred in 17.6% versus 15.7% of patients receiving Placebo versus Cocculine (p = 0.62) and severe vomiting in 10.8% versus 12.0% of patients receiving Placebo versus Cocculine (p = 0.72).

Four serious adverse events (SAEs), including 1 cutaneous papular eruption, 2 cases of anxiety/depression syndrome and 1 cholecystectomy for biliary colic, were reported but none were related to Cocculine. The overall incidence of AE (any type any grade) was similar between the 2 arms.

## Discussion

This study evaluates the potential clinical benefit of a complex homeopathic medicine for the control of CINV in a large homogenous randomized population of early breast cancer patients receiving uniform protocols of chemotherapy. Overall, adding thiscomplex homeopathic medicine to standard anti-emetic regimen did not improve the control of CINV in early breast cancer patients under chemotherapy regimen. FLIE nausea scores after the first chemotherapy course were 6.02 and 6.07 for placebo and Cocculine arms, respectively (p = 0.84, Table 3).

Our study was initially performed to confirm the efficacy of Cocculine in control of CINV in early breast cancer patients as reported by Genre *et al.,*[16]. However, our data showed no superiority of Cocculine over placebo. Several aspects of methodology may explain this discrepancy. Firstly, Genre *et al*. performed a small, single-centre study whereas more than 400 patients were enrolled in our multi-center clinical trial. Secondly, our prospective investigation was based on the FLIE score derived from a validated 18-item, patient-reported questionnaire. As CINV is frequently underestimated by caregivers, the FLIE questionnaire is an essential and better patient-reported tool to assess the functional impact of CT on patients’ daily lives. Relevant to our negative results, it should be noted that in the current study the percentage of patients with no or minimal impact of CINV on their daily life was particularly high (51% of patients in both placebo and Cocculine arms) and the incidence of severe nausea and vomiting were low (nausea: 17.6-15.7%, and vomiting 10.8-12%, Table 5). In contrast, Genre *et al*. have reported that 87.5% of patients enrolled in placebo arm have experienced nausea episodes during the first cycle of chemotherapy [16]. Of note, the emetyogenic properties of the chemotherapy used in our study as well as the standard anti-emetic treatment were similar. For additional comparison, the prevalence of CINV in previously reported clinical trials was around 65% (depending on chemotherapy regimen, and/or anti-emetic treatment used) [10,12,13]. Of note, one limitation of our study is the absence of data collection related to patients' belief as to group allocation (Cocculine or placebo), patient’s belief of homeopathy efficacy, comfort of homeopathy, pre-existing anxiety, history of alcohol consumption. Therefore, no comparison between groups for such patient-related factors, which may have influenced the development of CINV, was performed.

Considering that patients’ expectancies are largely influenced by the attention and information they received from clinicians, and are predictors, and, likely, contributing factors to the development of treatment-related emesis [20,21], we have followed 34 breast cancer patients during their 1^st^ cycle of chemotherapy (FAC50, FEC100 or TAC) in a post-study cohort. In contrast to patients enrolled in our Phase III, these patients were requested to complete a FLIE questionnaire without a recall on Day 5. Interestingly, at the end of the 1^st^ cycle, the proportion of patients with no or minimal impact of nausea on QoL (FLIE score >6) in this post-study cohort was only 31% (data not shown) versus 51 % in our Phase III population. Therefore, it is possible that extra attention provided to the patients enrolled in this study (i.e. with 5-day recall) may have change their perception of may be beneficial in terms of the occurrence and intensity of treatment-related side effects [6,22].

Our study has evaluated the effect of a complex homeopathic medicine in a large randomized study. This strategy was chosen for the following reasons: (i) first of all, the management of side effects related to conventional treatment need to be integrated in the routine of daily clinical practice thus not allowing time-consuming individual homeopathic prescription, (ii) secondly, the use of homeopathy with an individualized remedy is expensive and required the implication of the same experienced homeopath that is difficult to set up in multicenter trial and (iii) finally, individualized prescription is not easily compatible with double-blind randomized trials [23].

Although controversial, homeopathy is increasingly used worldwide as a complementary medicine and has been largely investigated in clinical trials [24-27]. However, no definitive conclusions can be drawn due to the low methodological quality of clinical trials, the small number of patients involved, lack of replication and presumed publication bias [28]. More and higher quality clinical trials, unbiased by belief or disbelief in the principles of homeopathy, need to be performed to assess the potential effect of such alternative medicines in controlling the side effects of cancer treatment [29].

## Conclusions

In conclusion, this double-blinded, placebo-controlled, randomised Phase III study showed that adding a complex homeopathic treatment to standard anti-emetic prophylaxis does not improve the control of CINV in early breast cancer patients.

## Competing interests

The authors declare that they have no competing interests. The study was partially funded by Boiron laboratories. The study treatments (Cocculine and placebo) were also provided by Boiron Laboratories. However, no representative from the funding sources participated at any stage of the trial, from either design to publication.

## Authors’ contributions

JP, AC H-B, TB; DC, J-P J, J-P G, CA, XP, IRC recruited and treated patients. DP, AB and IRC contributed to the design of the trial. DP and AB did the statistical analysis of the trial. All authors reviewed the manuscript before submission. All authors read and approved the final manuscript.

## Pre-publication history

The pre-publication history for this paper can be accessed here:

http://www.biomedcentral.com/1471-2407/12/603/prepub
